# Neonatal HDL Counteracts Placental Vascular Inflammation via S1P–S1PR1 Axis

**DOI:** 10.3390/ijms21030789

**Published:** 2020-01-25

**Authors:** Ilaria Del Gaudio, Sebastian Hendrix, Christina Christoffersen, Christian Wadsack

**Affiliations:** 1 Department of Obstetrics and Gynecology, Medical University of Graz, 8036 Graz, Austria; ilaria.del-gaudio@medunigraz.at (I.D.G.); hendrixsebastian@gmail.com (S.H.); 2 Department of Biomedical Sciences, University of Copenhagen, 1165 Copenhagen, Denmark; christina.christoffersen@regionh.dk; 3 Department of Clinical Biochemistry, Rigshospitalet, 2100 Copenhagen, Denmark

**Keywords:** neonatal high-density lipoprotein, vascular inflammation, feto-placental dysfunction, sphingosine-1-phosphate

## Abstract

Placental inflammation and dysfunction during pregnancy are associated with short- and long-term adverse outcomes for the offspring. However, the mechanisms of vascular protection at the feto-placental interface are still poorly investigated. The high-density lipoprotein (HDL) associated sphingosine-1-phosphate (S1P) has been described as a powerful anti-inflammatory complex. This study aimed to elucidate the role of cord blood-derived HDL (nHDL) in feto-placental endothelial dysfunction. Here, we report that the exposure of primary fetal placental arterial endothelial cell (fPAEC) to healthy nHDL-S1P attenuated the ability of TNFα to activate NF-κB signaling and increase the expression of pro-inflammatory markers. Moreover, the angiotensin II (AngII)-induced reactive oxygen species (ROS) production was blunted in the presence of nHDL, whereas it was preserved when the cells were preincubated with S1P receptor antagonists, suggesting that S1P accounts for the vascular protective function of nHDL at the feto-placental unit. These results highlight the importance of HDL and S1P metabolism and signaling in pregnancy pathophysiology.

## 1. Introduction

Abnormal placentation and failure of the maternal vasculature to adapt to pregnancy hemodynamics can result in the development of pregnancy-associated diseases such as gestational hypertension and pre-eclampsia (PE) [1,2]. For instance, PE affects 6–10% of all pregnancies worldwide and remains a leading cause of maternal and fetal morbidity and mortality [3,4]. Vascular inflammation and endothelial dysfunction are the major and closely interconnected hallmarks underlying these pathological conditions. The feto-placental endothelium is part of a complex barrier which separates maternal and fetal circulation and regulates the gas and nutrients exchange between mother and fetus. Because of its localization at the interface of the two circulations, the proper function of the feto-placental barrier is of crucial importance for fetal health and development.

High-density lipoproteins (HDLs) are potent anti-atherogenic mediators in adult human circulation due to their capability to remove cholesterol from peripheral tissues [5,6]. However, basic research and clinical studies suggest that the anti-inflammatory and endothelial protective function of HDL may play a much more important role in vascular health than cholesterol removal [7]. HDL possesses several beneficial pleiotropic properties which have been partially attributed to the action of the bioactive lipid sphingosine-1-phosphate (S1P), which is mainly carried through apolipoprotein M (ApoM) by the HDL particle in the circulation and, to a lesser extent, by serum albumin [8].

S1P is present at high concentrations in the lymphatic and circulatory systems (~1µM), whereas its tissue levels are relatively low (~75 pmol/mg) in physiological condition [9,10]. This vascular S1P gradient is crucial for the vascular homeostasis preservation. S1P can regulate endothelial function through specific G protein-coupled receptors (GPCRs) expressed on the vasculature. S1P receptor 1 (S1PR1) is highly abundant in endothelial cells and plays a pivotal role in vascular homeostasis [11]. Moreover, mice lacking the S1PR1 specifically in the endothelium exhibit a pro-inflammatory phenotype, suggesting the protective function of S1P-S1PR1 signaling in vascular pathology [12].

The role of free or HDL-associated S1P in vascular dysfunction has been predominantly studied in the context of atherosclerosis, diabetes or coronary artery disease (CAD) with controversial results [12,13,14,15,16,17]. However, nothing is known about the role of the HDL–S1P complex in placental vascular inflammation and dysfunction. In this study we aimed to investigate the role of cord blood-derived HDL (neonatal HDL, nHDL) in feto-placental dysfunction, focusing our attention on the fetal side of the circulation.

Our results clearly demonstrate that nHDL limits vascular inflammation by inhibiting NF-κB signaling and pro-inflammatory markers expression on the feto-placental vasculature. Moreover, the complex attenuates AngII-induced feto-placental dysfunction acting via S1P signaling. This is the first work which involves the use of human HDLs and isolated primary human placental endothelial cells to unravel the mechanisms of vascular dysfunction in placenta biology and pregnancy.

## 2. Results

### nHDL-S1P Complex Attenuates Inflammation and Dysfunction of the Feto-Placental Endothelium

**Figure 1:**
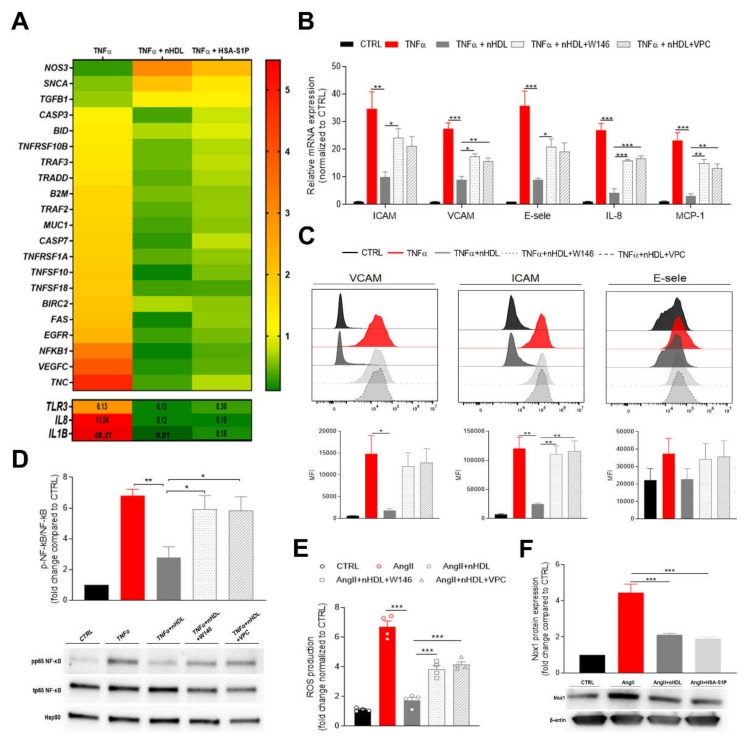
Cord blood derived high-density lipoprotein (nHDL)-bound S1P suppresses endothelial inflammatory markers and oxidative stress at the feto-placental vasculature. (**A**) Fetal placental arterial endothelial cells (fPAECs) were incubated with 10 ng/mL TNF-α in the presence of 800 µg/mL nHDL (~0.4 µmol/L S1P) or 1 µmol/L albumin-associated (has)-S1P for 6 h. Total RNA was analyzed by RT-PCR. Genes associated with preeclampsia and TNF-induced apoptosis, which were significantly regulated, as determined by multiple t-testing, are depicted in the heatmap as the average of *n* = 3 different isolations representing three biological replicates. Genes with values exceeding the scale shown in the upper panel are reported in the lower panel. (**B**) Changes in the expression of *VCAM*, *ICAM*, *E-selectin*, *IL-8* and *MCP-1* in fPAECs pre-treated with S1PRs inhibitors W146 and VPC23019 for 30 min before TNF-α and nHDL incubation for 6 h. Total RNA was analyzed by RT-PCR using TaqMan probes. Data were normalized to control and presented as mean ± SEM (*n* = 4). One-way ANOVA followed by Tukey’s multiple comparison. * *p* < 0.05; ** *p* < 0.01; *** *p* < 0.001. (**C**) fPAEC were treated as in (**B**) and analyzed by flow cytometry: VCAM, ICAM and E-selectin. Representative flow cytometry histograms (upper panel) and bar graphs showing the median fluorescence intensity (MFI) (lower panel). Data are presented as mean ± SEM (*n* = 3). One-way ANOVA followed by Tukey’s multiple comparison. * *p*<0.05; ** *p*<0.01. (**D**) Cells were serum-starved treated as in (B) for 1 h. Phosphorylation (p) of the NF-κB subunit p65 was analyzed by Western blot of total cell lysates. Hsp90 was used as loading control. Data were normalized to control and expressed as fold change ratio of phospho p65/ total p65 (mean ± SEM of *n* = 4 independent experiments). One-way ANOVA followed by Tukey’s multiple comparison. * *p* < 0.05; ** *p* < 0.01. (**E**) fPAECs were pre-treatment with S1PRs inhibitors (W146 and VPC23019 1 µmol/L for 30 min) and incubated with dichlorofluorescin diacetate (DCFDA) for 45 min followed by exposure to 1 µmol/L AngII in the presence of 800 µg/mL nHDL. Intracellular oxidation of was detected by fluorescence spectroscopy (Ex/Em, 295/529). Tert-butyl hydrogen peroxide (200 µmol/L) was used as a positive control. The bar chart shows the reactive oxygen species (ROS) production as fold change (mean ± SEM; *n* = 4) compared to control. One-way ANOVA followed by Tukey’s multiple comparison. *** *p* < 0.001. (**F**) fPAECs were serum starved and treated with 1 µmol/L AngII in the presence of 800 µg/mL nHDL or 1 µmol/L HSA-S1P for 6 h. Protein expression of Nox1 was analyzed by western blot in whole cell lysates. β-Actin served as loading control. Data are presented as mean ± SEM (*n* = 3). One-way ANOVA followed by Tukey’s multiple comparison. *** *p* < 0.001.

To study the role of nHDL-S1P or albumin-associated S1P (HSA-S1P) on placental vascular inflammation, we challenged primary human placental arterial endothelial cells (fPAEC) with TNF-α for 6 h and determined the changes in gene expression in the presence of the two complexes. We analyzed a panel of genes which are triggered by inflammation and are known to be associated with TNF-induced apoptosis and preeclampsia (Figure 1A). As expected, TNF-α strongly induced the expression of genes belonging to the TNF and TNF-receptor superfamily, such as TNFSF10, TNFSF18, TNFRSF1A and TNFRSF10. Genes involved in apoptosis (CASP3, CASP7, FAS and BID) were also upregulated. However, the presence of nHDL-S1P and HSA-S1P, although differentially effective, mitigated the expression of TNF-α-induced genes. Moreover, the mRNA of different effectors of the NF-κB signaling pathway, like TRADD, TRAF2, TRAF3, BRIC2, was upregulated upon TNF-α stimulation and downregulated in the presence of nHDL. Interestingly, the most pronounced increase in fold change was observed for the pro-inflammatory cytokines IL-1β, IL-8 and for TLR3, which are key mediators of inflammation in preeclampsia [18,19]. However, nHDL-S1P and HSA-S1P strongly abolished their transcription. By contrast, TNF-α markedly downregulated the expression of nitric oxide synthase 3 (NOS3), which is involved in the production of nitric oxide (NO) in the endothelium. Notably, nHDL was able to prevent this effect more efficiently than HSA-S1P, suggesting a carrier-dependent regulation of NOS3 expression.

Vascular inflammation promotes phenotypic changes in the endothelium characterized by the over-expression of adhesion molecules and the release of pro-inflammatory cytokines, which initiate and contribute to endothelial dysfunction. To further validate the anti-inflammatory effect of nHDL and explore the role of S1P signaling within this pathway, we pre-incubated fPAEC with a selective antagonist for S1PR1 (W146) or an antagonist for S1PR1/3 (VPC23019) [20], which are the receptors subtypes widely distributed in the cardiovascular system, then challenged fPAECs with TNF-α and examined the mRNA expression of different inflammatory markers (Figure 1B). The addition of nHDL to the culture medium markedly reduced the TNF-α-induced expression of intracellular adhesion molecule-1 (ICAM-1), vascular cell adhesion molecule-1 (VCAM-1), E-selectin, monocyte chemoattractant protein-1 (MCP-1) and interleukin 8 (IL-8). Strikingly, the inhibitors treatment restored inflammation, suggesting that the anti-inflammatory effect of the neonatal particle is partially mediated by S1P. These results were independently confirmed by flow cytometry analysis, which revealed that the surface expression of the adhesion molecules was decreased in the presence of nHDL (Figure 1C).

Among potential intracellular pathways involved in the regulation of endothelial inflammatory response, nuclear factor κB (NF-κB) represents a key player. Indeed, the NF-κB promoter controls the transcription of many pro-inflammatory genes, including adhesion molecules, chemokines and cytokines [21]. TNF-α stimulation of fPAEC increased NF-κB (p65) phosphorylation, which was significantly suppressed by co-incubation with nHDL (Figure 1D). Moreover, blockage of S1PR1 did not modify the endothelial inflammatory response. These observations corroborate the concept that nHDL exert its anti-inflammatory effect by delivering S1P to its receptors on the fetal endothelium.

To further explore the ability of nHDL to prevent the endothelial dysfunction, we exposed fPAEC to angiotensin II (AngII). It has been shown that AngII is a powerful stimulator of NADPH oxidase (Nox), which generates reactive oxygen species (ROS), thus triggering vascular inflammatory response [22]. AngII treatment remarkably increased ROS production, a response that was notably suppressed when nHDL was added to the cells (Figure 1E). Similarly to what we observed for the TNF-induced inflammatory response, the blocking of S1P signaling led to the reversal of nHDL’s capability of lowering ROS production. Furthermore, we demonstrated that nHDL and HSA-S1P decreased the abundance of Nox isoform 1 (Nox1), which is expressed in the feto-placental vasculature. These data suggest that the nHDL–S1P complex plays a significant role in AngII-induced placental dysfunction.

HDL-S1P content and signaling has been shown to be impaired in cardiovascular diseases and diabetes [23]. Indeed, HDL-associated S1P levels are reportedly lower in subjects with CAD or T2D [14,15]. Our proteomic data showed that nHDL from PE pregnancies have a lower ApoM content, suggesting decreased S1P levels associated with the particle (Figure S1).

## 3. Discussion

Vascular inflammation represents an early event in several cardiovascular and metabolic diseases. On the other hand, low-grade inflammation is a physiological process for maternal adaptation to pregnancy. However, the line between a normal and pathological inflammatory response in pregnancy is blurred and still not fully understood. In recent years, much research has been done to clarify the role of placental inflammation and its association with adverse effects on fetal development. Nonetheless, there is a lack of knowledge of the mechanisms of placental vascular regulation and function. Maternal systemic inflammation can affect placental physiology.

As feto-placental endothelium is continuous with the fetal circulation, any dysregulation of its function may lead to impaired fetal growth. Pathologies such as obesity-associated gestational diabetes, fetal growth restriction and preeclampsia (PE) have been associated with exacerbated inflammatory response and changes in feto-placental endothelium phenotype and vascular tone [24,25].

Human pregnancy is accompanied by hyperlipidemia (particularly during the last trimester), with a rise in triglycerides and cholesterol levels in HDL and LDL particles, to meet the cholesterol demand of the fetus [26]. LDL particles represent the major cholesterol carrier in the maternal circulation, where they also serve as a substrate for placental progesterone synthesis [27,28]. Conversely, in cord blood circulation the HDL particle is the cholesterol carrier [29], suggesting a specific and distinct role of the cord blood-derived particles compared to the adult ones.

The bioactive mediator S1P associated with the HDL particle is known to have a major impact on the physiology of the endothelium [30,31,32,33]. Several studies have shown that HDL and S1P are involved in the regulation of the endothelial inflammatory response in atherosclerosis [12,13]. However, the underlying mechanisms are still poorly understood in the context of pregnancy and placental pathophysiology.

Our study highlights the ability of neonatal HDL–S1P to suppress the inflammatory response and dysfunction of the feto-placental endothelium by reducing the expression of inflammatory markers and lowering the vascular oxidative stress. Increased levels of TNF-α in PE have been linked with endothelial activation and damage [34]. We could show that nHDLs, which represent a unique class of HDL circulating in the cord blood and in fetal circulation, as well as HSA-S1P, can affect gene regulation under inflammatory conditions. Although both complexes were able to suppress the expression of genes involved in cellular apoptosis and vascular dysfunction, nHDL were more efficient. The reason for this effect relies not only in the heterogeneity of the nHDL particle but presumably also in an S1P carrier-dependent regulation of inflammation [13]. However, we cannot draw any definitive conclusions in this context, since the concentration of S1P on the two carriers is different (~0.4 µmol/L S1P associated with nHDL versus 1 µmol/L S1P associated with HSA)

TNF-α triggers the expression of cytokines and cell adhesion molecules for leukocytes’ recruitment during vascular inflammation. We demonstrated that fPAEC exposed to nHDL had decreased the mRNA expression of these inflammatory markers, confirming the anti-inflammatory properties of the particle. Fourth, the pre-incubation of fPAEC with W146 (selective inhibitor of S1PR1) and VPC23019 (inhibitor of S1PR1/3), pre-empted the inhibitory effects of nHDL on TNF-α-induced adhesion molecule expression. Furthermore, the pharmacological inhibition of both receptors did not show any further effects via receptor 3, supporting the concept that S1PR1 is the main actor in the anti-inflammatory signaling. Using defined preparations of human HDL (HDL+ApoM with S1P or HDL-ApoM lacking S1P), Ruiz et al. elegantly showed that the increased expression of VCAM, ICAM and E-selectin was suppressed only by the HDL fraction containing S1P. In agreement with their work, we showed that the surface expression of VCAM and ICAM was inhibited in presence of nHDL. However, E-selectin expression was only affected at the transcriptional level. Several factors might contribute to the observed discrepancy in previous results, such as different experimental incubation times (4 h vs. 6 h), type of HDL (adult vs neonatal) and type of endothelium (human aortic endothelial cells vs human feto-placental arterial endothelial cells). Furthermore, we used a different experimental approach involving the use of S1PRs antagonists in the presence of the native particles.

NF-κB signaling is activated with increased oxidative stress and inflammation. Indeed, placentae from PE-women have an exaggerated activation of the pathway compared to healthy ones [35]. Galvani et al. reported that HDLs isolated from WT mice can blunt the TNF-α- induced NF-κB activation, whereas HDLs from ApoM KO mice are not effective to the same extent, suggesting that S1P is mediating the HDL effect [12]. Moreover, they showed that human HDL in human umbilical vein endothelial cells (HUVECs) can achieve the same result in a dose-dependent manner. However, in the case of human HDL, they did not confirm the link between S1P signaling and HDL’s capability of suppressing the activation of the NF-κB pathway. Our data corroborate the concept that human HDL can interfere with NF-κB signaling activation. In addition, we demonstrated a direct, S1P-dependent effect on the signaling cascade by using pharmacological inhibitors for S1PR1/3.

Disruption of the oxygen balance with concomitant increased ROS production plays an important role in inflammation-associated vascular cell damage [36]. Numerous studies have indeed reported an association between placental dysfunction, ROS production and the onset of hypertension in PE [37,38]. NAD(P)H oxidase is an important source of ROS within the vascular wall, and its activity is differentially regulated in pathophysiological processes. Tölle and co-workers demonstrated that HDL-associated lysosphingolipids inhibit the thrombin-induced NAD(P)H oxidase activity and ROS production in vascular smooth muscle cells (VSMCs) [39]. In the present study, we show that nHDL negatively regulates AngII-induced ROS production in fPAEC via S1P signaling. Furthermore, we demonstrated that nHDL and HSA-S1P can decrease the protein expression of the NAD(P)H oxidase catalytic subunit Nox1. These data provide a partial insight into the mechanism by which nHDL and S1P protect the endothelium from oxidative stress in the placenta.

Another novel observation was that PE is associated with reduced ApoM levels on nHDL. Although further functional studies are needed to link the reduction in ApoM, as well as S1P content, to nHDL dysfunction, our findings corroborate the concept that the nHDL–S1P complex plays a pivotal role in the maintenance of placental vascular homeostasis.

Despite our study clearly demonstrating the vasculoptotective effects of the nHDL–S1P complex on the feto-placental vasculature, some limitations should be noted. As proof-of-concept, TNF-α and AngII were used as vascular disruptor molecules in our experimental design in order to evaluate the capability of nHDL to attenuate placental endothelial inflammation and dysfunction. However, we cannot directly translate these findings to the complex inflammatory and hypertensive environment which characterizes PE. Thus, studies assessing how and whether PE affects the function of the placental endothelium as well as the functionality of the nHDL particles are warranted.

## 4. Materials and Methods

### 4.1. Study Population

Clinical characteristics of the subjects enrolled in the study are summarized in Table S1. Each individual in this study gave written informed consent. All experiments were performed in accordance with the protocols approved by the ethics committee of the Medical University of Graz (Vote no: 29-319 ex 16/17, approval date: 29/03/2017).

### 4.2. Isolation of Cord Blood-Derived Neonatal HDL and S1P Quantification

Neonatal cord blood plasma (*n* = 10 male and *n* = 10 female) were centrifuged from collected mixed (arterial and venous) umbilical cord blood samples, which were obtained immediately after delivery of the placentas. nHDLs were isolated by ultracentrifugation [40,41]. The purity of total HDL was assessed by measuring apolipoprotein composition, total protein and cholesterol. Subsequently, S1P concentration was determined by HPLC, as described elsewhere [8].

### 4.3. Isolation and Treatment of Primary Fetal Placental Arterial Endothelial Cells (fPAEC)

Term placentae from caesarean section and vaginal delivery were used within 20 min of delivery (*n* = 4). fPAECs were isolated from arterial chorionic blood vessels as described by Lang et al. [42]. Cells were treated as follows: 10 ng/mL TNF-α or 1 µmol/L AngII in the presence of 800 µg/mL nHDLs (which were isolated according to Section 2 and contain ~0.4 µmol/L S1P) or 1 µmol/L HSA-S1P (Avanti Polar Lipids, Alabaster, AL, USA). Selective inhibitor of S1PR1–W146 (1 µmol/L) (857390, Avanti Polar Lipids) and S1PR1/3 inhibitor VPC23019 (1 µmol/L) (4195, Tocris Bioscience, Bristol, UK), were used in the cell culture experiments. When cells were treated with W146 or with VPC23019, Na_2_CO_3_, (2-hydroxypropyl)-β-cyclodextrin and DMSO respectively, were added as a vehicle.

### 4.4. Quantitative Real-Time PCR (qPCR) and PrimePCR of fPAEC

Cells were seeded in 12 well plates at a density of 100,000 cells/well. After two days, medium was changed to serum-free EBM and cells were treated for 6 h as described in Section 4.3. Treatment was performed in triplicates. After treatment, cells were washed twice and harvested in 350 µl RLT Lysis buffer (Quiagen, Hilden, Germany) supplemented with 1% β-mercaptoethanol (Sigma Aldrich, St. Louis, MO, USA). Next, total RNA content was isolated using the RNeasy^®^ Mini Kit (Quiagen, Hilden, Germany) according to manufacturer’s instructions. Quantitative real-time PCR was performed on the CFX384 cycler (BioRad Technologies, Vienna, Austria) using TaqMan^®^ Gene Expression assays (Applied Biosystems, Thermo Fisher Scientific, Carlsbad, CA, USA) and TaqMan^®^ Universal PCR Master Mix (Applied Biosystems, Thermo Fisher Scientific, Carlsbad, CA, USA). Used TaqMan^®^ Gene Expression assays are listed in Table S2. PrimePCR panels for pre-eclampsia (Pre-eclampsia Tier 1 H384; BioRad Technologies, Vienna, Austria) and apoptosis (Apoptotic TNF Family pathways H384; BioRad Technologies, Vienna, Austria) were used according to manufacturer’s instructions. Relative quantification of gene expression was calculated by ∆∆*C*q method.

### 4.5. Western Blot

fPAEC were plated at a density of 200,000 cells/well in 6-well plates (Thermo Fisher Scientific). After two days in culture, cells were serum starved for 6 h and treated for 1 or 6 h as mentioned above. Thereafter, cells were collected in 50 μL RIPA lysis and extraction buffer (Sigma Aldrich) containing protease inhibitors (Roche, Basel, Switzerland). Total protein concentration was determined by bicinchoninic acid assay (BCA; Thermo Fisher Scientific). A total of 10 µg of total protein was loaded onto 4–20% SDS-PAGE gradient gels (BioRad Technologies) and resolved at 120 V for 1 h 10 min. Membranes were incubated with antibodies against phospho-p65 NF-κB (3033; Cell signaling, Danvers, MA, USA), total p65 NF-κB (8242; Cell signaling), Hsp90 (610418; BD Bioscience; New Jersey, NJ, USA), Nox1 (ab55831, Abcam, Cambridge, UK) and β-actin (ab6276, Abcam). All antibodies were diluted in 5% non-fat dry milk (BioRad Technologies, Vienna, Austria). Detection was carried out using SuperSignal^®^ Chemiluminescent Substrate (Thermo Fisher Scientific). Immunolabeling was visualized with the Fusion FX imaging system (Vilber Lourmat, Marne-la-Vallée, France) and band densitometry was performed using the Fusion© Software (Vilber Lourmat). Hsp90 and β-actin were used as housekeeping genes.

### 4.6. FACS of fPAECs

Cells were carefully harvested by using 1 mL accutase (PAA, Pasching, Austria). Detached cells were collected in 10 mL HBSS, counted and centrifuged for 4 min at 800 rpm. The obtained cell pellet was resuspended (1x10^6^ cells/mL) in staining buffer containing PBS (Thermo Fisher Scientific) supplemented with 0.1% bovine serum albumin (Sigma Aldrich) and 20 mmol/L EDTA (Thermo Fisher Scientific). Subsequently cells were incubated for 30 min at 4°C in the dark with the following antibodies: CD106-APC (305809, BioLegend, San Diego, CA, USA), CD54-Pacific Blue (322715, BioLegend) and CD62E-PE (322605, BioLegend). Unstained cells were used as a control. For each sample, at least 10,000 cells were counted. Cell sorting was performed on a CytoFLEX flow cytometer (Beckman Coulter, Brea, CA, USA) using the associated CytEXpert software (Beckman Coulter) for setting the gates and analysing data.

### 4.7. Reactive Oxygen Species (ROS) Assay

Cells were seeded in a dark-wall, clear-bottom, 96-well microplate (Costar^®^; Corning Inc., New York, NY, USA) at a density of 20,000 cells/well. 100 µL of DCFDA solution (10 µmol/L in HBSS) (Abcam) were added to each well and cells were stained for 45 min at 37 °C in the dark. Subsequently, cells were washed and treated with 1 µmol/L AngII (Sigma Aldrich) in the presence of 800 µg/mL nHDL with or without inhibitors for 6 h. A total of 200 µmol/L tert-butyl hydrogen peroxide (TBHP; Abcam) was used as positive control. All treatment compounds were diluted in phenol red free DMEM (Gibco, Thermo Fisher Scientific) without supplements. Fluorescent intensity of oxidized dichlorofluorescein (DCF) was measured at Ex/Em = 485/535 in end point mode (FLUOstar Optima; BMG Labtech, Offenburg, Germany).

### 4.8. Shotgun Proteomics by LC-MS/MS

Proteomic analysis were performed as previously described [43]. Relative protein abundance between samples was calculated based on the number of spectral counts (SpCs) of the total peptide.

### 4.9. Statistical Analysis

Graph Pad Prism 7 Software (GraphPad Software Inc., San Diego, USA) was used for all the statistical calculations and graph plotting. *t*-test or one-way ANOVA, including Tukey post-hoc, were performed if two or more groups were compared, respectively. For PrimePCR data, differences in experimental groups were evaluated by multiple Students’ *t*-test using the Holm–Sidak method for multiple comparison correction. *p*-values below 0.05 were considered statistically significant.

## 5. Conclusions

In conclusion, our study defines S1P signaling as a key mediator of nHDL’s anti-inflammatory effect on feto-placental endothelium. Furthermore, we provided evidence that the nHDL–S1P complex is a powerful regulator of ROS formation, thereby protecting the endothelium from oxidative stress and preserving placental vascular function. Thus, S1P and HDL metabolism and signaling might represent an attractive therapeutic target in pregnancy-associated diseases.

## Supplementary Materials

Supplementary materials can be found at https://www.mdpi.com/1422-0067/21/3/789/s1.

## Abbreviations

ApoM	Apolipoprotein M
GPCR	G protein-coupled receptors
S1PR	Sphingosine-1-phosphate receptor
HSA	Human serum albumin
TNFα	Tumor necrosis factor alpha
AngI	Angiotensin II
fPAEC	Human primary fetal placental endothelial cell
ROS	Reactive oxygen species
Nox1	NADPH oxidase 1
ICAM	Intracellular adhesion molecule
VCAM	Vascular cell adhesion molecule
E-sele	Endothelial-leukocyte adhesion molecule
IL-8	Interleukin 8
MCP-1	Monocyte chemoattractant protein 1

## References

[1] Fisher S.J. (2015). Why is placentation abnormal in preeclampsia?. Am. J. Obstet. Gynecol..

[2] Granger J. (2001). Pathophysiology of pregnancy-induced hypertension. Am. J. Hypertens..

[3] Backes C.H., Markham K., Moorehead P., Cordero L., Nankervis C.A., Giannone P.J. (2011). Maternal Preeclampsia and Neonatal Outcomes. J. Pregnancy.

[4] Duley L. (2009). The Global Impact of Pre-eclampsia and Eclampsia. Semin. Perinatol..

[5] Barter P., Gotto A.M., LaRosa J.C., Maroni J., Szarek M., Grundy S.M., Kastelein J.J.P., Bittner V., Fruchart J.-C. (2007). HDL Cholesterol, Very Low Levels of LDL Cholesterol, and Cardiovascular Events. N. Engl. J. Med..

[6] Terasaka N., Yu S., Yvan-Charvet L., Wang N., Mzhavia N., Langlois R., Pagler T., Li R., Welch C.L., Goldberg I.J. (2008). ABCG1 and HDL protect against endothelial dysfunction in mice fed a high-cholesterol diet. J. Clin. Invest..

[7] Navab M., Reddy S.T., Van Lenten B.J., Fogelman A.M. (2011). HDL and cardiovascular disease: Atherogenic and atheroprotective mechanisms. Nat. Rev. Cardiol..

[8] Christoffersen C., Obinata H., Kumaraswamy S.B., Galvani S., Ahnstrom J., Sevvana M., Egerer-Sieber C., Muller Y.A., Hla T., Nielsen L.B. (2011). Endothelium-protective sphingosine-1-phosphate provided by HDL-associated apolipoprotein M. Proc. Natl. Acad. Sci..

[9] Berdyshev E.V., Gorshkova I.A., Garcia J.G., Natarajan V., Hubbard W.C. (2005). Quantitative analysis of sphingoid base-1-phosphates as bisacetylated derivatives by liquid chromatography–tandem mass spectrometry. Anal. Biochem..

[10] Edsall L.C., Spiegel S. (1999). Enzymatic Measurement of Sphingosine 1-Phosphate. Anal. Biochem..

[11] Liu Y., Wada R., Yamashita T., Mi Y., Deng C.-X., Hobson J.P., Rosenfeldt H.M., Nava V.E., Chae S.-S., Lee M.-J. (2000). Edg-1, the G protein–coupled receptor for sphingosine-1-phosphate, is essential for vascular maturation. J. Clin. Invest..

[12] Galvani S., Sanson M., Blaho V.A., Swendeman S.L., Obinata H., Conger H., Dahlbäck B., Kono M., Proia R.L., Smith J.D. (2015). HDL-bound sphingosine 1-phosphate acts as a biased agonist for the endothelial cell receptor S1P 1 to limit vascular inflammation. Sci. Signal..

[13] Ruiz M., Frej C., Holmér A., Guo L.J., Tran S., Dahlbäck B. (2017). High-Density Lipoprotein–Associated Apolipoprotein M Limits Endothelial Inflammation by Delivering Sphingosine-1-Phosphate to the Sphingosine-1-Phosphate Receptor 1. Arterioscler. Thromb. Vasc. Biol..

[14] Frej C., Mendez A.J., Ruiz M., Castillo M., Hughes T.A., Dahlbäck B., Goldberg R.B. (2017). A Shift in ApoM/S1P Between HDL-Particles in Women With Type 1 Diabetes Mellitus Is Associated With Impaired Anti-Inflammatory Effects of the ApoM/S1P Complex. Arterioscler. Thromb. Vasc. Biol..

[15] Sattler K., Gräler M., Keul P., Weske S., Reimann C.M., Jindrová H., Kleinbongard P., Sabbadini R., Bröcker-Preuss M., Erbel R. (2015). Defects of High-Density Lipoproteins in Coronary Artery Disease Caused by Low Sphingosine-1-Phosphate Content Correction by Sphingosine-1-Phosphate—Loading. J. Am. Coll. Cardiol..

[16] Keul P., Lucke S., von Wnuck Lipinski K., Bode C., Gräler M., Heusch G., Levkau B. (2011). Sphingosine-1-Phosphate Receptor 3 Promotes Recruitment of Monocyte/Macrophages in Inflammation and Atherosclerosis. Circ. Res..

[17] Fernández-Pisonero I., Dueñas A.I., Barreiro O., Montero O., Sánchez-Madrid F., García-Rodríguez C. (2012). Lipopolysaccharide and Sphingosine-1-Phosphate Cooperate To Induce Inflammatory Molecules and Leukocyte Adhesion in Endothelial Cells. J. Immunol..

[18] Szarka A., Rigo J.J., Lazar L., Beko G., Molvarec A. (2010). Circulating cytokines, chemokines and adhesion molecules in normal pregnancy and preeclampsia determined by multiplex suspension array. BMC Immunol..

[19] Chatterjee P., Weaver L.E., Doersch K.M., Kopriva S.E., Chiasson V.L., Allen S.J., Narayanan A.M., Young K.J., Jones K.A., Kuehl T.J. (2012). Placental Toll-Like Receptor 3 and Toll-Like Receptor 7/8 Activation Contributes to Preeclampsia in Humans and Mice. PLoS ONE.

[20] PETERS S., ALEWIJNSE A. (2007). Sphingosine-1-phosphate signaling in the cardiovascular system. Curr. Opin. Pharmacol..

[21] Baker R.G., Hayden M.S., Ghosh S. (2011). NF-κB, Inflammation, and Metabolic Disease. Cell Metab..

[22] Brasier A.R., Recinos A., Eledrisi M.S. (2002). Vascular Inflammation and the Renin-Angiotensin System. Arterioscler. Thromb. Vasc. Biol..

[23] Levkau B. (2015). HDL-S1P: Cardiovascular functions, disease-associated alterations, and therapeutic applications. Front. Pharmacol..

[24] Boeldt D.S., Bird I.M. (2017). Vascular adaptation in pregnancy and endothelial dysfunction in preeclampsia. J. Endocrinol..

[25] Knock G.A., McCarthy A.L., Lowy C., Poston L. (1997). Association of gestational diabetes with abnormal maternal vascular endothelial function. BJOG An Int. J. Obstet. Gynaecol..

[26] Herrera E., Ortega-Senovilla H. (2014). Lipid Metabolism During Pregnancy and its Implications for Fetal Growth. Curr. Pharm. Biotechnol..

[27] Edison R.J., Berg K., Remaley A., Kelley R., Rotimi C., Stevenson R.E., Muenke M. (2007). Adverse Birth Outcome Among Mothers With Low Serum Cholesterol. Pediatrics.

[28] Chatuphonprasert W., Jarukamjorn K., Ellinger I. (2018). Physiology and Pathophysiology of Steroid Biosynthesis, Transport and Metabolism in the Human Placenta. Front. Pharmacol..

[29] Nagasaka H., Chiba H., Kikuta H., Akita H., Takahashi Y., Yanai H., Hui S.-P., Fuda H., Fujiwara H., Kobayashi K. (2002). Unique character and metabolism of high density lipoprotein (HDL) in fetus. Atherosclerosis.

[30] Theilmeier G., Schmidt C., Herrmann J., Keul P., Schäfers M., Herrgott I., Mersmann J., Larmann J., Hermann S., Stypmann J. (2006). High-Density Lipoproteins and Their Constituent, Sphingosine-1-Phosphate, Directly Protect the Heart Against Ischemia/Reperfusion Injury In Vivo via the S1P 3 Lysophospholipid Receptor. Circulation.

[31] Obinata H., Hla T. (2012). Sphingosine 1-phosphate in coagulation and inflammation. Semin. Immunopathol..

[32] Nofer J.-R., Bot M., Brodde M., Taylor P.J., Salm P., Brinkmann V., van Berkel T., Assmann G., Biessen E.A.L. (2007). FTY720, a Synthetic Sphingosine 1 Phosphate Analogue, Inhibits Development of Atherosclerosis in Low-Density Lipoprotein Receptor–Deficient Mice. Circulation.

[33] Del Gaudio I., Sreckovic I., Zardoya-Laguardia P., Bernhart E., Christoffersen C., Frank S., Marsche G., Illanes S.E., Wadsack C. (2020). Circulating cord blood HDL-S1P complex preserves the integrity of the feto-placental vasculature. Biochim. Biophys. Acta Mol. Cell Biol. Lipids.

[34] Meekins J.W., McLaughlin P.J., West D.C., McFadyen I.R., Johnson P.M. (1994). Endothelial cell activation by tumour necrosis factor-alpha (TNF-alpha) and the development of pre-eclampsia. Clin. Exp. Immunol..

[35] Vaughan J.E., Walsh S.W. (2012). Activation of NF-κB in Placentas of Women with Preeclampsia. Hypertens. Pregnancy.

[36] Forrester S.J., Kikuchi D.S., Hernandes M.S., Xu Q., Griendling K.K. (2018). Reactive Oxygen Species in Metabolic and Inflammatory Signaling. Circ. Res..

[37] Sedeek M., Gilbert J.S., LaMarca B.B., Sholook M., Chandler D.L., Wang Y., Granger J.P. (2008). Role of Reactive Oxygen Species in Hypertension Produced by Reduced Uterine Perfusion in Pregnant Rats. Am. J. Hypertens..

[38] Siddiqui I.A., Jaleel A., Tamimi W., Al Kadri H.M.F. (2010). Role of oxidative stress in the pathogenesis of preeclampsia. Arch. Gynecol. Obstet..

[39] Tölle M., Pawlak A., Schuchardt M., Kawamura A., Tietge U.J., Lorkowski S., Keul P., Assmann G., Chun J., Levkau B. (2008). HDL-Associated Lysosphingolipids Inhibit NAD(P)H Oxidase-Dependent Monocyte Chemoattractant Protein-1 Production. Arterioscler. Thromb. Vasc. Biol..

[40] Sattler W., Mohr D., Stocker R. (1994). Rapid isolation of lipoproteins and assessment of their peroxidation by high-performance liquid chromatography postcolumn chemiluminescence. Methods Enzymol..

[41] Holzer M., Kern S., Trieb M., Trakaki A., Marsche G. (2017). HDL structure and function is profoundly affected when stored frozen in the absence of cryoprotectants. J. Lipid Res..

[42] Lang I., Schweizer A., Hiden U., Ghaffari-Tabrizi N., Hagendorfer G., Bilban M., Pabst M.A., Korgun E.T., Dohr G., Desoye G. (2008). Human fetal placental endothelial cells have a mature arterial and a juvenile venous phenotype with adipogenic and osteogenic differentiation potential. Differentiation.

[43] Sreckovic I., Birner-Gruenberger R., Obrist B., Stojakovic T., Scharnagl H., Holzer M., Scholler M., Philipose S., Marsche G., Lang U. (2013). Distinct composition of human fetal HDL attenuates its anti-oxidative capacity. Biochim. Biophys. Acta Mol. Cell Biol. Lipids.

