# Supplementary material for: Neonatal HDL Counteracts Placental Vascular Inflammation via S1P–S1PR1 Axis
Source: Int J Mol Sci. 2020 Jan 25;21(3):789. doi: 10.3390/ijms21030789 (PMC7037016; doi:10.3390/ijms21030789)
Supplement: Supplementary file 1 [file ijms-21-00789-s001.pdf]

International Journal of *Molecular Sciences*

MDPI

Supplementary Material

Neonatal HDL counteracts placental vascular inflammation via S1P-S1PR1 axis

Ilaria Del Gaudio [1], Sebastian Hendrix [1], Christina Christoffersen [2,3] and Christian Wadsack [1,*]

[1] Department of Obstetrics and Gynecology, Medical University of Graz, Graz, Austria; ilaria.del-gaudio@medunigraz.at; hendrixsebastian@gmail.com; christian.wadsack@medunigraz.at
[2] Department of Biomedical Sciences, University of Copenhagen, Copenhagen, Denmark
[3] Department of Clinical Biochemistry, Rigshospitalet, Copenhagen, Denmark; christina.christoffersen@regionh.dk
* Correspondence: christian.wadsack@medunigraz.at; Tel.: +43-316-385-81074 (C.W.)

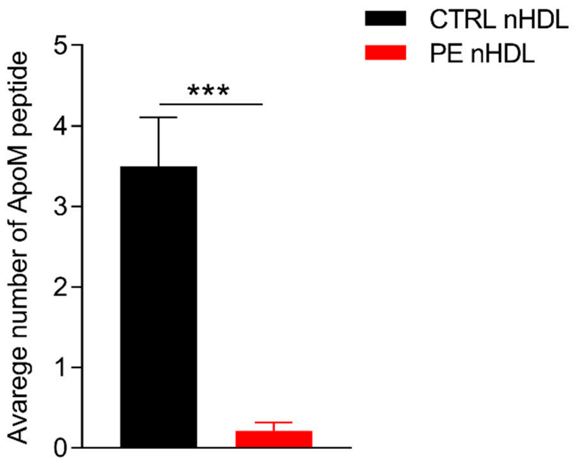

Supplementary Figure S1. ApoM content in neonatal HDL from control and PE pregnancies. ApoM peptide comparison between control (n=10) and PE (n=9) nHDL obtained by LC-MS/MS. Data are presented as mean± SEM. Statistical significance was assessed by Student's t-test; ***p≤0.001.

Supplementary Table S1. Characteristics of subjects whose cord blood was used for nHDL isolation. Abbreviations: BMI=body-mass index; PI = fetal ponderal index. Fetal PI is comparable to BMI in adult subjects but relates weight to the cubed body length. SBP = systolic blood pressure; DBP = Diastolic blood pressure All data are presented as mean±SD. Statistical significance was assessed by Student's t-test. **p≤0.001,***p≤0.0001.

	**Control Group (*n* = 20)**	**PE Group (*n* = 9)**
Age	29 ± 3	28±2.3
BMI	24 ± 2.9	24±2.5
Gestational week	38 ± 1	36.6±2.8
Placental weight (g)	654 ± 105.4	410.8±110.2**
Fetal PI (kg/m3)	2.5 ± 0.7	2.4±0.3
SBP (mmgH)	112±8.1	155±9.4***
DBP (mmgH)	71±7.2	105±6.7***

Supplementary Table S2. TaqMan® Gene Expression assays used for quantitative real-time PCR.

Target	TaqMan® Assay ID	Supplier
Intercellular adhesion molecule 1 (ICAM1)	Hs00164932_m1	Thermo Fisher Scientific
Vascular cell adhesion molecule 1 (VCAM1)	Hs01003372_m1	Thermo Fisher Scientific
Interleukin 8 (IL-8)	Hs00174103_m1	Thermo Fisher Scientific
Monocyte chemoattractant protein 1 (MCP1)	Hs00234140_m1	Thermo Fisher Scientific
CD62 antigen-like family member E (E-Selectin)	Hs00174057_m1	Thermo Fisher Scientific
TATA-box binding protein (TBP)	Hs00427620_m1	Thermo Fisher Scientific
Proteasome subunit beta 6 (PSMB6)	Hs00382586_m1	Thermo Fisher Scientific
Hypoxanthine phosphoribosyltransferase1 (HPRT1)	Hs02800695_m1	Thermo Fisher Scientific

© 2020 by the authors. Licensee MDPI, Basel, Switzerland. This article is an open access article distributed under the terms and conditions of the Creative Commons Attribution (CC BY) license (http://creativecommons.org/licenses/by/4.0/).
